# The Association of COVID-19 Infection with Abnormal Thyroid Function Tests

**DOI:** 10.51894/001c.143925

**Published:** 2025-09-30

**Authors:** Safa Abdalla

**Affiliations:** 1 Henry Ford Warren

## 20

### BACKGROUND

COVID-19 has been reported to affect the thyroid gland. Most studies in this area are either small or uncontrolled, and very few considered the concurrent use of thyroid test-altering medications or the role of age and sex.

### OBJECTIVE

To investigate the association between abnormal thyroid tests and COVID-19 infection, accounting for baseline thyroid function and medications, and exploring the role of age and sex.

### METHODS

We included 162 COVID-19-positive admissions in Henry Ford Warren Hospital in December 2023 where TSH levels were measured. A random sample of COVID-negative admissions was created by including one that was closest in admission time to each COVID-19-positive admission. Data included demographics, BMI, known thyroid disease, level of care during admission, steroid use during admission, and use of thyroid function-altering medications before admission. We compared those factors between the two groups using the Chi-squared test and the Mann-Whitney U test. As the TSH level’s distribution was skewed, we compared the median TSH level between the two groups using the Mann-Whitney U test, accounting for variables that were not balanced between the two groups.

### RESULTS

Of the total sample, 55.9% were male, 65.7% were 65 years or older, 23.5% had a known history of thyroid disease, and 38.0% were on thyroid test-altering medications other than thyroid disease treatment. 21.3% were on a critical care floor, and 12.7% received steroids before TSH measurement. The two groups had a statistically significant difference in median age (p < 0.001) and the percentage of known thyroid dysfunction (p = 0.018), but none for the other tested variables. Among those with no known thyroid dysfunction, median TSH was 1.64mU/L and 1.45mU/L in the COVID and non-COVID groups respectively (p = 0.054). In a stratified analysis, differences in median TSH between the two groups were not statistically significant among males and females and among the different age groups (less than 45, 45-64, 65+).

### CONCLUSION

Our study did not show a significant difference in TSH levels between COVID-19 and non-COVID admissions. This could reflect a less aggressive COVID-19 illness course in 2023.

